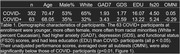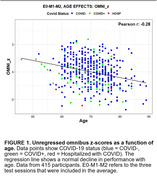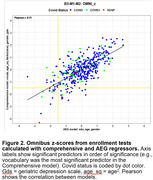# Does COVID‐19 Infection Accelerate Age‐Related Cognitive Decline?

**DOI:** 10.1002/alz.091557

**Published:** 2025-01-03

**Authors:** David L. Woods, Kathleen Hall, Peter Pebler, Garrett Williams, Timothy J Herron, Michael Blank, Kristin Geraci, David K Johnson, Krista Schendel, Sandy J. Lwi, Jas M. Chok, Omar Kahly, Maria G Spinelli, Isabella Jaramillo, Juliana Baldo

**Affiliations:** ^1^ Neurobehavioral Systems, Inc, Berkeley, CA USA; ^2^ University of Chicago, Chicago, IL USA; ^3^ Veterans Affairs Northern California Health Care System, Martinez, CA USA; ^4^ UC Davis Alzheimer’s Disease Center, Walnut Creek, CA USA; ^5^ Palo Alto University, Palo Alto, CA USA

## Abstract

**Background:**

Similar neuroimaging abnormalities are reported in post‐COVID patients and patients with AD. Moreover, COVID‐19 and Alzheimer’s Disease (AD) share genetic vulnerabilities and have similar cognitive symptoms of COVID (e.g., memory impairment, mental fatigue, anosmia, etc.). These findings raise the concern that COVID infection may increase the risk that older patients will develop Alzheimer’s disease.

**Method:**

Self‐reported COVID histories were recorded from 415 older participants (mean age 70.1 years), including 310 participants longitudinally tested beginning in 2022. The effects of COVID on cognitive performance were assessed with 32 computerized subtests of the California Cognitive Assessment Battery (CCAB). Omnibus z‐scores were averaged over 70 test measures at enrollment, and again at 6‐month and 18‐month post‐enrollment in the 310‐participant sample.

**Result:**

Significant demographic differences were seen between COVID+ and COVID‐ groups (Table 1). COVID+ patients performed worse than COVID‐ patients overall (Figure 1, *t(85) = ‐2.80, p<0.01*) and when analyzed with a conventional model using Age, Education, and Gender (AEG) as regressors (*t(85) = ‐3.07, p<0.005*, Figure 2). However, inter‐group differences failed to reach significance (*t(85) = ‐0.97, NS)* when analyzed with a comprehensive 15‐factor model that factored out additional demographic influences including vocabulary, comorbidities, depression, anxiety, functional status, and race. In contrast, COVID infection severity (estimated from augmented WHO scores) correlated significantly with omnibus z‐scores when analyzed with the comprehensive model (*r = ‐0.30, t(64) = ‐2.52, p<0.02*) but correlations failed to reach significance when analyzed with the conventional (AEG) model.

The effect of incidental “breakthrough” COVID infections in participants (97.35% were vaccinated prior to retest) was analyzed at 6‐months (38 cases) and 18‐months (50 cases). Breakthrough infections prior to 6‐month retest were paradoxically associated with improved 6‐month Omnibus scores *(t(49) = 2.74, p < 0.01*), while no significant differences were observed for breakthrough infections prior to the 18‐month retest (*t(90) = 0.95, NS*).

**Conclusion:**

These preliminary results suggest that infections with Wild Type and Delta variants early in the COVID pandemic produced cognitive deficits among older participants, particularly among patients hospitalized with COVID. In contrast, post‐inoculation breakthrough infections caused no significant decline in cognitive performance.